# Maternal Intake of Cow’s Milk during Lactation Is Associated with Lower Prevalence of Food Allergy in Offspring

**DOI:** 10.3390/nu12123680

**Published:** 2020-11-28

**Authors:** Mia Stråvik, Malin Barman, Bill Hesselmar, Anna Sandin, Agnes E. Wold, Ann-Sofie Sandberg

**Affiliations:** 1 Department of Biology and Biological Engineering, Food and Nutrition Science, Chalmers University of Technology, 412 96 Gothenburg, Sweden; mia.stravik@chalmers.se (M.S.); malin.barman@chalmers.se (M.B.); 2 Institute of Environmental Medicine, Unit of Metals and Health, Karolinska Institutet, 171 77 Stockholm, Sweden; 3 Department of Paediatrics, Institute of Clinical Sciences, Sahlgrenska Academy, University of Gothenburg, 405 30 Gothenburg, Sweden; bill.hesselmar@vgregion.se; 4 Department of Clinical Science, Pediatrics, Sunderby Research Unit, Umeå University, 901 87 Umeå, Sweden; anna.sandin@umu.se; 5 Department of Infectious Diseases, Institute of Biomedicine, Sahlgrenska Academy, University of Gothenburg, 413 90 Gothenburg, Sweden; agnes.wold@microbio.gu.se

**Keywords:** nutrition, pregnancy, lactation, food intake, food allergy, atopic eczema, asthma, erythrocytes, breast milk, dietary biomarkers

## Abstract

Maternal diet during pregnancy and lactation may affect the propensity of the child to develop an allergy. The aim was to assess and compare the dietary intake of pregnant and lactating women, validate it with biomarkers, and to relate these data to physician-diagnosed allergy in the offspring at 12 months of age. Maternal diet during pregnancy and lactation was assessed by repeated semi-quantitative food frequency questionnaires in a prospective Swedish birth cohort (*n* = 508). Fatty acid proportions were measured in maternal breast milk and erythrocytes. Allergy was diagnosed at 12 months of age by a pediatrician specialized in allergy. An increased maternal intake of cow’s milk during lactation, confirmed with biomarkers (fatty acids C15:0 and C17:0) in the maternal blood and breast milk, was associated with a lower prevalence of physician-diagnosed food allergy by 12 months of age. Intake of fruit and berries during lactation was associated with a higher prevalence of atopic eczema at 12 months of age. Our results suggest that maternal diet modulates the infant’s immune system, thereby influencing subsequent allergy development.

## 1. Introduction

Allergy is one of the most common chronic diseases in childhood, affecting up to 30% of children in industrialized countries [1]. Food allergy and atopic eczema may appear already during the first year of life, while allergic asthma and allergic rhinoconjunctivitis usually appear at school age or later. Atopic eczema is a strong risk factor for sensitization to environmental allergens and subsequent asthma [2]. For instance, sensitization to egg protein during infancy predicts a high risk of subsequent development of allergic asthma [3].

While the etiology of allergies is not yet completely understood, it appears to reflect complex interactions of genetic and various environmental and lifestyle factors. Maternal diet during pregnancy and lactation have been discussed as potential lifestyle factors that can modify the risk of allergy in the offspring [4]. For instance, maternal intake of specific food items such as fish and dairy products has been associated with lower risk of allergic outcomes in the offspring [5,6]. On the contrary, intake of margarine and vegetable oil has been associated with a higher risk of offspring eczema [7]. A wide range of dietary factors have been identified as immunoregulatory in humans [8,9]. For instance, vitamin D, which is highly abundant in fish and fortified dairy products, is known to have an extensive impact on the immune system [10]. Another example is unsaturated fatty acids, commonly consumed as margarine or vegetable oils, which are known to dampen the immune system by suppressing the activation of T cells, particularly Th1 cells [11,12,13].

Even though several studies have been conducted on the role of maternal diet on offspring allergy, more studies that relate to allergies diagnosed by physicians using strict criteria, as opposed to studies that merely report sensitization or parent-reported allergies, are needed. Dietary intake is difficult to access with questionnaires, regardless of method. Therefore, objective measures such as the use of fatty acids in blood and breast milk as dietary biomarkers, provide confirmation of the actual intake.

The aim of the current study was to assess and compare the dietary intake of pregnant and lactating women using questionnaires, validate it with dietary biomarkers in erythrocytes and breast milk, and relate these data to physician-diagnosed allergy in the offspring at 12 months of age.

## 2. Materials and Methods

### 2.1. Study Population

The Nutritional impact on Immunological maturation during Childhood in relation to the Environment (NICE) study, which is a birth cohort study based at the Sunderby Hospital in northern Sweden, is designed to investigate the influences of lifestyle factors during pregnancy and early in life on immune cell maturation and allergy development [14]. In brief, pregnant women who were able to communicate effectively in Swedish were informed about the study at their first visit to the local maternity clinic in Gestational Weeks 10–12. Recruitment took place in connection with a routine ultrasound in Gestational Week 18; 637 families were included in the NICE cohort between February 2015 and March 2018. Eighteen of the families participated in the study with two subsequent pregnancies, three women gave birth to twins, five foetuses died before birth, and one woman had a late miscarriage after inclusion in the study, resulting in 652 live-born children being included in the cohort. In the statistical analyses, only the first sibling was included and twins were excluded.

This study was conducted in accordance with the Helsinki Declaration and was approved by the Regional Ethical Review Board in Umeå, Sweden (2013/18–31M, 2015–71–32). To be able to participate, all the women and their partners had to sign a written consent form, both for themselves and for their child. All participants were informed about their right to withdraw from the study at any point and to have their data removed.

### 2.2. Dietary Assessment

Three similar web-based semi-quantitative food frequency questionnaires (Meal-Q1, Meal-Q2 and Meal-Q3) were used to assess food intake during the previous month. The questionnaires were sent by email to the participants at Gestational Week 34, 1 month postpartum, and 4 months postpartum. Reminders were transmitted by email 1 and 2 weeks later to those who did not respond to the original email, with the second reminder being combined with a text message sent by mobile phone. The response rates for the three food frequency questionnaires were 94%, 93%, and 93%, respectively.

The original Meal-Q was developed by researchers at the Karolinska Institute and has been validated against a web-based, 7-day weighted food diary [15,16]. For the NICE study, this original version was revised to include the collection of detailed information regarding intake of heavy metals, sugars, probiotics, and fat quality, as previously described [17].

Food intake was reported as frequencies, ranging from 1–3 times/month to ≥5 times/day. In cases of no intake or intake less than once per month, the participants were instructed to leave the question blank. Meal-Q has a meal-based and interactive format, i.e., only those who report consumption of a certain food will receive follow-up questions regarding, for example, the use of low-calorie products, fat quality, and consumed amounts. Depending on the number of follow-up questions, the Meal-Q comprises 102–174 questions. The Meal-Q is a semi-quantitative questionnaire and contains follow-up questions on specific amounts of food items consumed. The participants were asked to specify their intakes from protein sources (meat, chicken, fish), carbohydrate sources (rice, potato, pasta etc.), and vegetables/salad in their main meals, using images that depict five different portion sizes. In addition, the consumed number of bread and cheese slices and the amount of candy and chocolate were specified.

Intake of macro and micronutrients were calculated based on the reported intake frequencies and portion sizes reported in the Meal-Q and did not include supplement use. The nutritional calculations were done by the research group that developed the questionnaires, as previously described in detail [15,16].

The intake frequencies were converted into intake in times per week. When the intake frequency was defined as an interval, the mean was calculated such that a frequency of 1–2 times/week was set to 1.5. The newly created variable was further converted into intake in gram per day, using either the above-mentioned reported intake levels or, when no levels were specified, standard portions from the Swedish Food Composition Database [18]. Food groups were created, summarizing the daily intake in gram per day of the included food items (see Supplementary Table S1).

### 2.3. Allergy Diagnosis

Diagnosis of allergic diseases during the first year of life was evaluated at 12 months of age by the study pediatrician specialized in allergology, according to predefined protocols. A total of 539 infants participated in the examination.

Atopic eczema was diagnosed according to William’s criteria [19,20,21].

Food allergy was defined as an immediate or late-onset reaction to intake of a specific food with prompt clinical improvement once the food allergen was removed from the diet. Except from when the first reaction was an acute severe reaction, the diagnosis was supported by provocation causing similar symptoms from the same organ. Sensitization or specific IgE antibodies against the specific food supported the diagnosis in some cases, but was not mandatory for diagnosis.

Asthma was diagnosed based on either documented wheezing between infections, a persistent wheeze for at least 4 weeks, a period of wheezing during an infection with concomitant allergic disease, or three episodes of wheezing during an infection, without concomitant allergic disease.

In total, 36 infants were diagnosed with atopic eczema (33 were included in the present study), 35 with asthma (33 were included in the present study), and 43 with food allergy (39 were included in the present study). When infants with food allergy were followed-up between 12 and 24 months of age, 21 of the 39 infants showed symptoms at provocation and were considered to have an ongoing food allergy also between 1 and 2 years of age in the statistical analyses in this study.

Allergic disease within the family was assessed at the 12-month follow-up with a structured interview with the parents, conducted by the pediatrician specialized in allergy. Allergic disease within the family was defined as having a parent or sibling with one or more of the following diagnoses; atopic eczema, food allergy, allergic rhinoconjunctivitis, and/or asthma with treatment.

### 2.4. Analysis of Fatty Acids in Erythrocytes

The total fatty acid composition was assessed in erythrocytes collected from the mothers at Gestational Week 28 and at 4 months postpartum. The analysis of fatty acids in erythrocytes is described in detail in Supplementary Materials. Briefly, 50 µL erythrocytes were mixed with 10 µg internal standard (methyl tricosanoate, C23:0 methyl ester) and 1.8 mL acetyl chloride-MeOH solution 10% (*v*/*v*) fortified with butylated hydroxytoluene (2.78 µg/mL). After methylation of the fatty acids during incubation at 70 °C for 1 h, the methyl esters were extracted with n-hexane, evaporated, and redissolved in 200 µL hexane before injected into the gas chromatography with flame ionization detector (GC-FID) for analysis. For details about the GC-FID system and the oven program please see Supplementary Materials. A pool of erythrocytes from five donors was aliquoted and stored at −80 °C and used as a quality control sample. The standard GLC-462 mixed fatty acid methyl esters (Nu-Chek Prep, Elysian, MN, USA) were used for the external standard calibration. Data were acquired using the Thermo Fisher Scientific Xcalibur ver. 4.3 software (Thermo Scientific, Waltham, MA, USA). Twenty-two fatty acids were quantified in each sample: 14:0, 15:0, 16:0, 16:1 n-7, 17:0, 18:0, 18:1 n-7, 18:1 n-9, 18:2 n-6, 18:3 n-3, 20:0, 20:1 n-11, 20:2 n-9, 20:3 n-6, 20:4 n-6, 20:5 n-3, 22:0, 22:4 n-6, 22:5 n-3, 22:6 n-3, 24:0, 24:1 n-9. The concentration of each fatty acid was calculated using a standard curve of the external standard with the internal standard C23:0 added. The proportion of specific fatty acids is expressed as the area of the particular fatty acid, relative to the concentration of all 22 fatty acids.

### 2.5. Analysis of Fatty Acids in Breast Milk

The concentrations of fatty acids in the total lipid fraction of breast milk samples collected from the mothers 1 and 4 months postpartum were analyzed by gas chromatography after conversion to methyl esters [22]. The analysis of fatty acids in breast milk is described in detail in Supplementary Materials. Briefly, 100 µL breast milk samples were mixed with 50 µg internal standard (fatty acid 19:0), 1 mL toluene, and 1 mL acetyl chloride-MeOH solution 10% (*v*/*v*). After methylation of the fatty acids during incubation at 70 °C for 2 h, the methyl esters were extracted with petroleum ether and MilliQ-water, evaporated, dissolved in 1 mL isooctane, and separated in a gas chromatography-mass spectrometry system (5975C; Agilent Technologies Inc., Santa Clara, CA, USA). A pool of breast milk from four donors was used as a quality control sample that was analyzed after every tenth sample to control the performance of the gas chromatography mass spectrometry (GC-MS) system. The standard set of GLC-463 mixed fatty acid methyl esters (Nu-Chek Prep) was used as an external standard for peak evaluation. Data were acquired using the Agilent MassHunter™ software. Thirty-three fatty acids were quantified in each sample: 10:0, 12:0, 14:0, 15:0, 16:0, 17:0, 18:0, 20:0, 22:0, 24:0, 14:1 n-5, 15:1, 16:1 n-7, 17:1 n-7, 18:1 n-7, 18:1 n-9, 19:1 n-9, 20:1 n-9, 22:1 n-9, 20:1 n-15, 18:3 n-3, 20:3 n-3, 20:5 n-3, 22:3 n-3, 22:5 n-3, 22:6 n-3, 18:2 n-6, 18:3 n-6, 20:2 n-6, 20:3 n-6, 20:4 n-6, 22:2 n-6, and 22:4 n-6. The concentration of each fatty acid was calculated using the concentration of the internal standard 19:0. The proportions of specific fatty acids were expressed as the concentration of the particular fatty acid relative to the concentration of all 33 fatty acids.

### 2.6. Selection of Subjects

Women who had live-born babies, singleton pregnancies, completed any of the three Meal-Q questionnaires, and had a reported energy intake within the range of 500–4000 kcal/day, were included in the current study. Given the primary outcome of the present study, only infants with an allergy examination at 12 months were included. In addition, to investigate at 1 month postpartum and 4 months postpartum the association between diet and allergy development, only women who lactated during the first month or the first four months, respectively, were included. In addition, for those participating with two pregnancies, only the first pregnancy was included.

The maternal diet during pregnancy have already been described for the NICE cohort in general terms [17]. The previous publication examined the associations between maternal characteristics and maternal dietary intake during pregnancy in 567 of the women in the NICE cohort. However, in the current study, the dietary regimen both during pregnancy and lactation is described and examined in relation to allergy development in offspring at 12 months of age. The current study includes 508 of the 567 women, due to other inclusion criteria (i.e., data from a 12-month follow-up visit).

### 2.7. Data Analysis

For the data analysis, IBM SPSS ver. 26 (IBM, New York, NY, USA) and R ver. 3.6.2 (R Foundation for Statistical Computing, Vienna, Austria) software packages were used. Pearson’s Chi-square and Fisher’s exact test were conducted to assess differences between allergic and non-allergic children with regard to background characteristics. To visualize the associations between maternal food intake and offspring allergic disease, unsupervised hierarchical cluster analyses were conducted and are presented as heatmaps together with partial Spearman’s correlations. The cluster analysis automatically structures the variables and places correlated variables next to each other.

Owing to the exploratory approach used, no adjustments were made for multiple testing. Spearman correlations were conducted between reported intake of cow’s milk products and fatty acid proportions in breast milk and in maternal erythrocytes.

### 2.8. Confounders

All analyses were adjusted for the pre-selected confounders: any allergy within family, siblings, season of birth, and total energy intake. Allergy within the family was added as yes or no and included allergy among the mother and/or father and/or siblings. Information regarding older siblings were collected with a questionnaire sent out to both parents in gestational week 18, asking about number of children living in the home full time. In seven cases, information regarding siblings were not available from the questionnaire. In these cases, information regarding parity was collected from the medical journal and used as a proxy for siblings. Regarding season of birth, children were divided into two groups depending on birth month: dark season (October to March) or bright season (April to September). The confounder adjusted analyses are shown in the main text while the unadjusted analyses are found in the supplementary files.

### 2.9. Reverse Causation

Symptoms of allergy in the offspring might result in elimination of the suspected allergen from the lactating mother’s diet. To take such reverse causation into account, data from questionnaires sent out monthly during the child’s first year of life were used. In these questionnaires, parents were asked to report if the child has had any symptoms related to food intake during the past month, and if so, which food item was suspected of causing these symptoms. For 13 individuals, data on early allergic symptoms were missing from the monthly questionnaires, so they were contacted by email and telephone retrospectively.

To take reverse causation into account, children with allergic symptoms before four months of age and diagnosed allergy were investigated further. Among these, dietary changes of the mothers were analyzed. In secondary analyses, children with allergic symptoms resulting in maternal avoidance of that specific food (i.e., change from any intake to 0 g/day) were excluded.

## 3. Results

**Figure 1:**
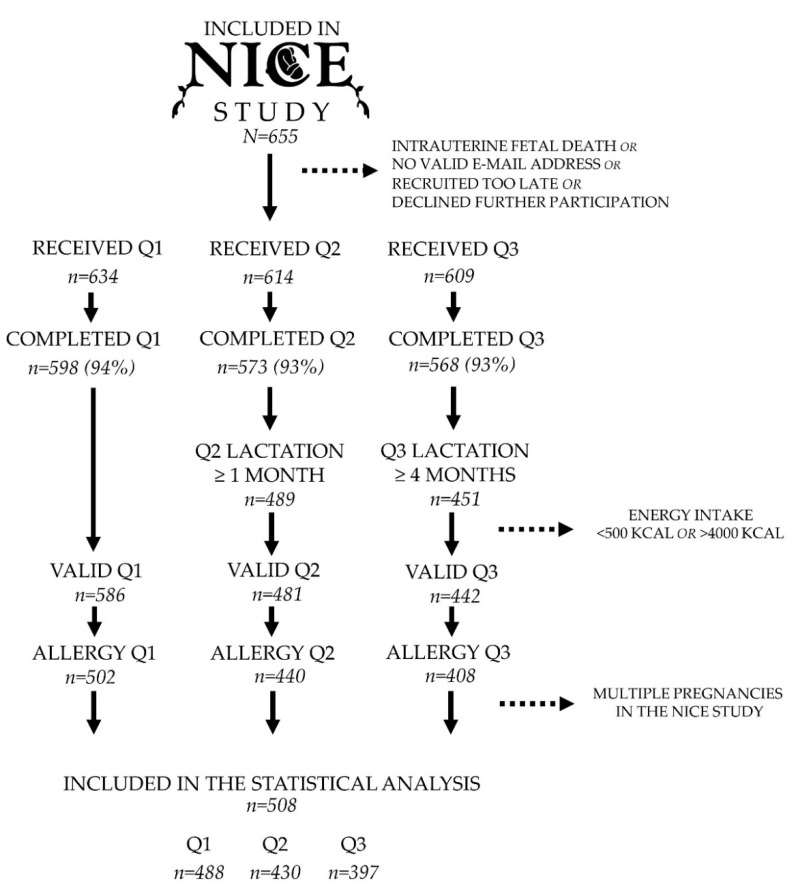
Flowchart of inclusion and exclusion in the present study. Dietary data were collected at three separate time-points: Gestational Week 34 (Q1), 1 month postpartum (Q2), and 4 months postpartum (Q3). All the families were invited to a follow-up visit at 12 months of age (Allergy Q1, Allergy Q2, and Allergy Q3) with a pediatrician specialized in allergy.

After the exclusion of twin-births, miscarriages or intrauterine fetal deaths, short duration of lactation (<1 month for diet 1 month postpartum and <4 months for diet 4 months postpartum), energy outliers (average daily intake <500 kcal or >4000 kcal), multiple pregnancies within the cohort, and no follow-up visit at 12 months, a total of 508 mother-child couples were included in the statistical analyses (Figure 1).

The median age of the 508 included mothers was 30 years (25th–75th percentile, 27–34). The majority had >12 years of education and BMI within the normal range at registration to the maternity clinic in early pregnancy (median, 24.3; 25th–75th percentile, 22.1–27.7).

The prevalence of different allergic manifestations at 12 months of age were 7.7% for food allergy, 6.5% for atopic eczema and 6.5% for asthma. Among the 39 children with food allergy, 8 were allergic solely to egg, 15 solely to cow’s milk, 5 solely to fish, and 1 solely to other. Ten children were allergic to multiple food items: 6 to egg and cow’s milk; 2 to egg, cow’s milk, and soy; 1 to egg and soy; 1 to egg and peanut.

Differences in characteristics between the children within the different diagnostic groups and the non-allergic children (i.e., non-allergic, non-sensitized, and non-asthmatic), are presented in Table 1. Maternal allergy (i.e., ongoing atopic eczema, food allergy, allergic rhinoconjunctivitis, and/or medicated asthma) was a strong predictor for offspring atopic eczema (64% vs. 37%, *p* = 0.002) and food allergy (59% vs. 37%, *p* = 0.007), and 97% of the children with atopic eczema had any allergic heredity (from parent and/or sibling), as compared to 67% of the non-allergic children (*p* < 0.001). There were more boys in the food allergy group than in the non-allergic group (62% vs. 44%, *p* = 0.033). Children with asthma more often had: fathers who smoked (14% vs. 2%, *p* = 0.017); shorter duration of breastfeeding (30% vs. 10% <4 months, *p* = 0.003); higher birthweight (16% vs. 4% with a birthweight >4.500 g, *p* = 0.019); and any sibling living at home full time (67% vs. 49%, *p* = 0.049), than the non-allergic children. No other significant differences were identified.

### 3.1. Food and Beverage Intake during Pregnancy and Lactation

Maternal intake of food items (in gram per day) at the three dietary assessment time-points are presented in Table 2. Specific food items included in each food group are specified in Supplementary Table S1. The intake of cow’s milk (fresh, pasteurized), dairy products (cow’s milk and cow’s milk products such as yoghurt, cheese, and sour cream), fruit and berries, and offal and yoghurt decreased successively from pregnancy until 4 months postpartum. The intake levels of grains rich in fiber (i.e., whole grain pasta, brown rice, soft grain bread, and crispbread) were higher during pregnancy than during lactation. Energy-adjusted intake of food items are presented in Supplementary Table S2.

The use of low-fat dairy products in our cohort was low. Instead, a great majority (90%) consumed cow’s milk with a fat percent between 1.5–3.0% fat.

### 3.2. Intake of Nutrients during Pregnancy and Lactation

Maternal intake of macronutrients and micronutrients per day during the three dietary assessment time-points, is presented in Table 3. The energy intake decreased gradually from pregnancy to 4 months postpartum, paralleled by reduced consumption of carbohydrates, more specifically, monosaccharides, fiber and sucrose, indicating an overall higher dietary intake during pregnancy than during lactation. The intake of total fat was lowest at 4 months postpartum, which can be explained by a lower intake of saturated fat and trans fat. Regarding micronutrients, highest intake of vitamin C, vitamin B6, and folate was seen during pregnancy, while the intake of iodine increased successively from pregnancy to 4 months postpartum. Energy-adjusted intake of macronutrients and micronutrients are presented in Supplementary Table S3.

### 3.3. Maternal Food Intake and Allergy in the Offspring

**Figure 2:**
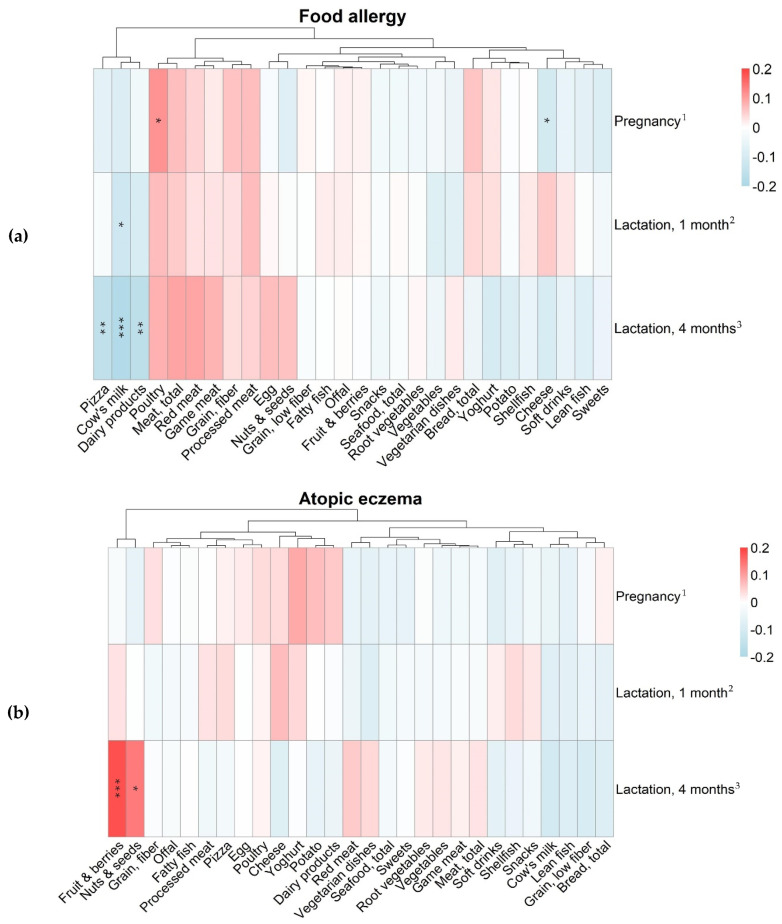

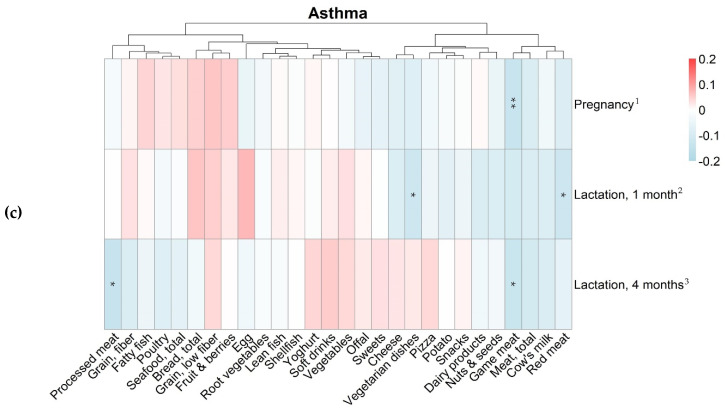
Heatmaps of correlations between reported maternal food intake (g per day) during pregnancy, lactation 1 month, and lactation 4 months, and offspring food allergy (**a**), atopic eczema (**b**) and asthma (**c**) during the first year of life. Associations between offspring allergy and maternal food intake were analyzed with partial Spearman’s correlation and all analyses were adjusted for any allergy within family, siblings, season of birth, and total energy intake. Definition of each dietary variable is presented in Supplementary Table S1. The magnitude of each correlation is denoted with a color, whereby red color indicates a positive correlation and blue color represents a negative correlation. Darker shades of these two colors indicate stronger correlations. Significant associations are denoted as: * *p* < 0.05, ** *p* < 0.01, and *** *p* < 0.001. Spearman’s rank correlation coefficients and *p* -values for significant associations are listed in Supplementary Table S4 (confounder adjusted) and Supplementary Table S5 (crude). For associations between allergy and food intake during lactation, only mothers who were breastfeeding at 1 and 4 months, respectively, were included in the analyses. Number of included subjects in the three figures are: (**a**) ^1^ n_tot_ = 409 and n_allergic_ = 38, ^2^ n_tot_ = 366 and n_allergic_ = 34, ^3^ n_tot_ = 338 and n_allergic_ = 30. (**b**) ^1^ n_tot_ = 403 and n_allergic_ = 32, ^2^ n_tot_ = 363 and n_allergic_ = 31, ^3^ n_tot_ = 335 and n_allergic_ = 27. (**c**) ^1^ n_tot_ = 402 and n_allergic_ = 31, ^2^ n_tot_ = 358 and n_allergic_ = 26, ^3^ n_tot_ = 332 and n_allergic_ = 24.

Associations between maternal food intake and offspring allergy were analyzed with partial Spearman’s rank correlation test with and without adjustment for any allergy within family, siblings, season of birth, and total energy intake. Results from the confounder adjusted tests are presented as heatmaps in Figure 2 and the Spearman’s rank correlation coefficients and *p*-values for the corresponding significant associations are listed in Supplementary Table S4. Results from the unadjusted Spearman’s rank correlation tests are shown in Supplementary Table S5. Details of what is included in each dietary variable can be found in Supplementary Table S1.

As can be seen in Figure 2a, higher intake of dairy products, primarily at four months postpartum, was associated with lower prevalence of food allergy. In addition, a higher intake of pizza at four months postpartum, cheese during pregnancy, and a lower intake of poultry during pregnancy was associated with lower prevalence of food allergy.

Since early allergic symptoms to specific foods in the weaning child might lead to elimination of these specific food products from the lactating mother’s diet, secondary analyses were conducted in which children with allergic symptoms resulting in maternal avoidance (i.e., changing from any consumption to 0 g/day) of dairy products at one and four months were excluded (*n* = 1 and *n* = 5, respectively). Higher maternal intake of cow’s milk during lactation was still significantly associated with lower prevalence of food allergy (1 month: rho_adj_ = −0.109, *p*_adj_ = 0.038; 4 months: rho_adj_ = −0.140, *p*_adj_ = 0.011). Hence, reverse causation cannot explain the associations found between cow’s milk products and offspring food allergy in our cohort.

Secondary analyses were also conducted on those children who had continuous reactions to food at provocation after 1 year of age (*n* = 21). The association between higher intake of cow’s milk during lactation and lower prevalence of offspring food allergy, remained statistically significant despite the loss of power (1 month, *n* = 18: rho_adj_ = −0.156, *p*_adj_ = 0.004; 4 months, *n* = 14: rho_adj_ = −0.154, *p*_adj_ = 0.006).

In Figure 2b, associations between maternal diet and offspring atopic eczema are presented. Similar to the findings reported for food allergy, strongest associations were found at four months postpartum, where higher maternal intake of fruit and berries and nuts and seeds was associated with higher prevalence of atopic eczema. Secondary analyses revealed that the association found for total fruit and berries was driven solely by banana and fruit soup, which *per se* were associated with atopic eczema (banana: rho_adj_ = 0.176, *p*_adj_ = 0.001; fruit soup: rho_adj_ = 0.132, *p*_adj_ = 0.016).

In contrast to food allergy and atopic eczema, the impact of maternal diet on offspring asthma did not seem to change remarkedly over time (Figure 2c). A higher intake of game meat during pregnancy, vegetarian dishes and red meat at one month postpartum, and processed meat and game meat at four months postpartum was associated with lower prevalence of asthma.

### 3.4. Maternal Intake of Nutrients and Allergy in the Offspring

**Figure 3:**
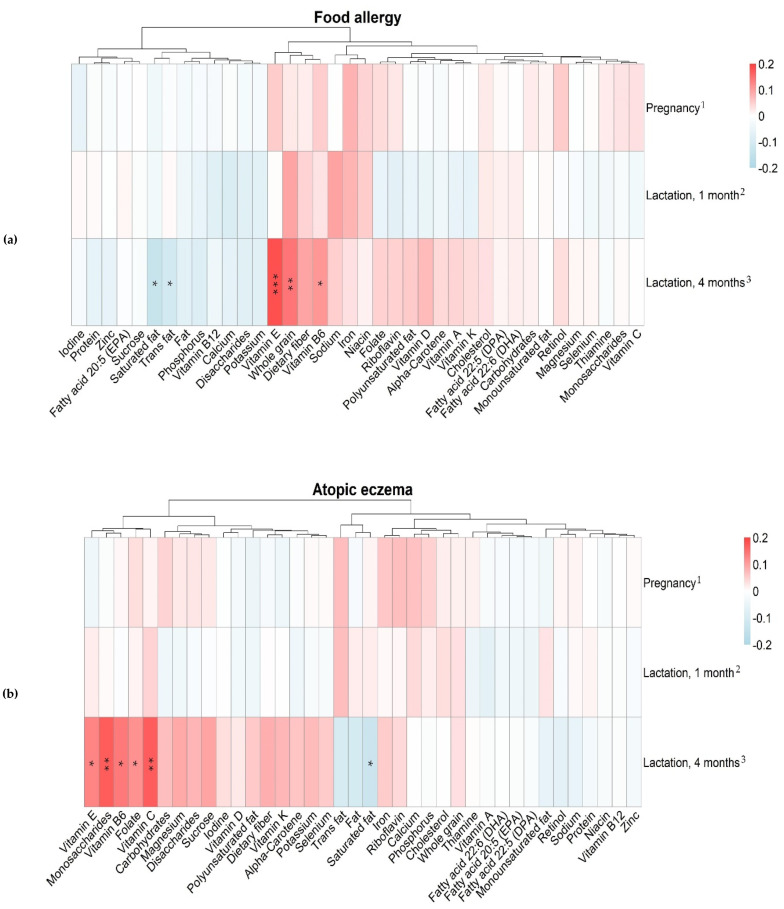

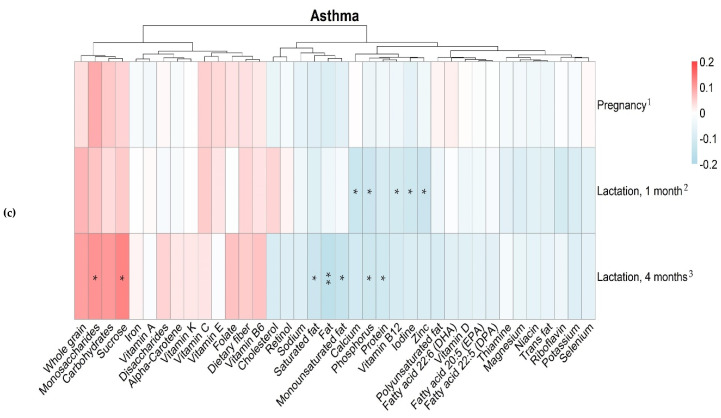
Heatmaps of correlations between reported maternal nutrient intake from dietary sources during pregnancy, lactation 1 month, and lactation 4 months, and offspring food allergy (**a**), atopic eczema (**b**) and asthma (**c**) at 12 months. Associations between offspring allergy and maternal nutrient intake were analyzed with partial Spearman’s correlation and adjusted for any allergy within family, siblings, season of birth, and total energy intake. The magnitude of each correlation is denoted with a color, whereby red color indicates a positive correlation and blue color represents a negative correlation. Darker shades of these two colors indicate stronger correlations. Significant associations are denoted as: * *p* < 0.05, ** *p* < 0.01, and *** *p* < 0.001. Spearman’s rank correlation coefficients and *p* -values for significant associations are listed in Supplementary Table S6 (confounder adjusted) and Supplementary Table S7 (crude). For associations between allergy and nutrient intake during lactation, only mothers who were breastfeeding at 1 and 4 months, respectively, were included in the analyses. Number of included subjects in the three figures are (**a**) ^1^ n_tot_ = 409 and n_allergic_ = 38, ^2^ n_tot_ = 366 and n_allergic_ = 34, ^3^ n_tot_ = 338 and n_allergic_ = 30. (**b**) ^1^ n_tot_ = 403 and n_allergic_ = 32, ^2^ n_tot_ = 363 and n_allergic_ = 31, ^3^ n_tot_ = 335 and n_allergic_ = 27. (**c**) ^1^ n_tot_ = 402 and n_allergic_ = 31, ^2^ n_tot_ = 358 and n_allergic_ = 26, ^3^ n_tot_ = 332 and n_allergic_ = 24.

Confounder adjusted associations between maternal nutrient intake and offspring allergy are presented in Figure 3. Spearman’s rank correlation coefficients and *p*-values for significant associations are listed in Supplementary Table S6 (confounder adjusted) and Supplementary Table S7 (crude).

As can be seen in Figure 3a, higher intake at four months postpartum of saturated fat and trans fat was associated with lower prevalence of food allergy. On the contrary, higher intake of vitamin E, whole grain, and vitamin B6 was associated with higher prevalence of offspring food allergy.

Similar to the findings for food allergy, higher maternal intake of saturated fat at four months postpartum was associated with lower prevalence of atopic eczema, while higher intake of vitamin E and vitamin B6 was associated with higher prevalence of atopic eczema (Figure 3b). In addition, higher intake of vitamin C, folate, and monosaccharides was associated with higher prevalence of atopic eczema.

Regarding the impact of maternal nutrient intake on asthma prevalence (Figure 3c), higher intake of calcium, phosphorus, vitamin B12, iodine, and zinc at one month postpartum were associated with lower prevalence of asthma. At four months postpartum, a higher intake of saturated fat, monounsaturated fat, total fat, phosphorus, and total protein was associated with a lower prevalence of asthma, while a higher intake of monosaccharides and sucrose was associated with a higher prevalence of asthma.

### 3.5. Fatty Acid Proportions in Maternal Erythrocytes and Breast Milk in Relation to Diet

To validate our dietary data and the finding that maternal intake of cow’s milk and dairy products was negatively associated with food allergy, fatty acid compositions of maternal erythrocyte membranes and breast milk were analyzed. Pentadecanoic acid (15:0) and heptadecanoic acid (17:0), which are synthesized in the cow’s rumen by the bacterial flora and, therefore, commonly used as specific biomarkers for dairy intake, were analyzed in breast milk sampled 1 and 4 months postpartum [23], as well as in maternal erythrocytes obtained at Gestational Week 28 and at 4 months postpartum (Table 4). Intake of dairy products, cow’s milk, and cheese correlated significantly with the proportion of pentadecanoic acid (15:0) and heptadecanoic acid (17:0) in breast milk. The correlations were weaker for fatty acid proportions in erythrocytes, but during lactation, both fatty acids correlated significantly with the proportions in erythrocytes (Table 4). Analyses were also conducted on energy-adjusted intakes, with similar results (data not shown).

We further analyzed the associations between proportion of pentadecanoic acid (15:0) and heptadecanoic acid (17:0) in breast milk and maternal erythrocytes in relation to allergy in the offspring. Pentadecanoic acid (15:0) in breast milk at 4 months postpartum was significantly associated with lower prevalence of physician-diagnosed food allergy in the offspring (rho_adj_ = −0.151, *p*_adj_ = 0.017).

## 4. Discussion

The overall aim of this study was to investigate the associations between maternal diet during pregnancy and lactation and physician-diagnosed allergy in the offspring during the first year of life. We found a significant association between a high maternal consumption of cow’s milk and cow’s milk products during lactation and lower prevalence of food allergy in the offspring. Further, higher maternal consumption of fruit and berries during lactation was significantly associated with increased prevalence of atopic eczema in the offspring.

The association between a protective effect of maternal intake of cow’s milk and offspring food allergy was further strengthened by the findings that intake of cow’s milk and cow’s milk products correlated with the proportions of pentadecanoic acid and heptadecanoic acid in breast milk and that proportions of pentadecanoic acid in breast milk in turn was associated with lower prevalence of offspring food allergy. In addition, a Finnish birth cohort study have previously reported maternal intake of cow’s milk during pregnancy to be protective against physician-diagnosed food allergy, more specifically allergy to cow’s milk protein in the offspring [5].

When the phenomenon of reverse causation was examined, a higher maternal intake of cow’s milk during lactation was still significantly associated with lower prevalence of food allergy in the offspring. Thanks to the thorough assessment of allergy, secondary analyses could also be conducted on children with ongoing food allergy after 1 year of age. Higher maternal intake of cow’s milk during lactation was significantly associated also with less ongoing food allergy after 1 year of age.

In Sweden, cow’s milk is mainly consumed as fresh, pasteurized milk with a fat percent between 0.5–3.0% and must be fortified with vitamin D until it reaches a level of 0.95–1.10 µg/100 g. Intake of vitamin D has previously been associated with reduced risk of asthma/wheeze [6] but with an increased risk of food allergy in the offspring [24]. In our study, we found no association of vitamin D intake to any of these allergies. Instead, a possible explanation for the beneficial effects of cow’s milk relates to the fat composition of these products [25], which typically have high concentrations of saturated fats and ruminant trans fats. Based on intakes from the nutritional calculations, we found that saturated fat and trans fats were significantly associated with lower prevalence of both food allergy and atopic eczema. In addition, we found that the intake of vitamin E, which is commonly found in products that are high in unsaturated fat but low in saturated fat, such as vegetable oils, was associated with higher prevalence of food allergy and atopic eczema.

By increasing the proportions of saturated fats in the diet, the intake of unsaturated fat is naturally decreased, given that the individual is in energy balance. It is essential that the immune system is stimulated during early life to ensure the development of immune tolerance and prevent allergy development. We have previously hypothesized that saturated fat is, not per se but through its competition with unsaturated fatty acids [26], beneficial in terms of allergy prevention [27,28]. Unsaturated fats are known to dampen the immune system by suppressing the activation of T cells and they may deprive the infantile immune system of the necessary activating stimuli during a crucial window of development. Another possible explanation for the beneficial influence of cow’s milk is its content of bioactive proteins and peptides. More specifically, its content of proline-rich polypeptides (PRP) have been found to enhance T-cell maturation [29] and osteopontin to modulate immune function and stimulate Th1/Th2 shifting [30]. Furthermore, maternal diet-derived food antigens in human milk in combination with immunoglobulins may provide a tolerogenic environment explaining the reduced risk of food allergy [31].

We also show a highly significant association between higher total intake of fruit and berries during lactation, 4 months postpartum, and higher prevalence of atopic eczema in the offspring. Fruit and berries, especially dried varieties, are a rich source of monosaccharides and vitamin C, which we also show to be associated with a higher prevalence of atopic eczema. However, previous studies regarding these associations have been inconclusive [4,6,32,33]. To investigate in greater depth the association noted for total fruit and berry intake, we conducted secondary analyses in which we separated the individual food items included in the collective group of fruit and berries. This analysis revealed significant associations solely between atopic eczema and maternal intake of banana and fruit soup. Banana, depending on its level of ripeness, and fruit soup, which usually contains potato starch or corn starch, are rich in resistant starch. Therefore, the effects seen for fruit may be due to its high content of resistant starch, which is in accordance with a previous study reporting an increased risk of physician-diagnosed eczema in offspring at 12 months of age when there was maternal intake of resistant starch during pregnancy [34]. On the other hand, even though the association between fruit and berries and atopic eczema is strongly significant, one might argue that our results are surprising since resistant starch and dietary fiber is favorable for the gut microbiome and might enhance oral tolerance, which may decrease allergy risk [35]. To verify our results, further studies with validated biomarkers for fruit intake related to eczema development are needed.

In addition to our findings regarding cow’s milk and fruit and berries, we also demonstrate significant associations between offspring allergy and maternal intake of poultry, pizza, nuts and seeds, vegetarian dishes, and different meat products such as game meat. We have previously shown that the consumption of some of these food items is associated with lifestyle factors such as education, age, and residency [17]. Based on the limited literature supporting these findings in the context of allergy development, these associations may therefore reflect an impact of lifestyle on allergy risk, rather than an impact of the diet per se. Since these associations are of low statistical significance, it is also important to keep in mind the risk of false findings due to multiple testing. In addition, the intake of different food items is closely related to each other, which makes it difficult to draw conclusions regarding a single food item.

Major strengths of the present study are that allergy was physician-diagnosed based on strict criteria and that all the children met with the same pediatrician specialized in allergy. Furthermore, the collection of biologic specimens was extensive, and our results are strengthened by the measurements of biomarkers in erythrocytes and breast milk. Another strength of this study is the high response rates (93–94%) to the dietary questionnaires.

Due to the observational design of our study, we cannot confirm causality, as there is always a risk of residual confounding. However, the comprehensive dataset in the NICE cohort allowed us to account for a high number of possible confounders. Another limitation is that the dietary data were quantified based on intake frequency and a description of a general portion size. To collect even more accurate dietary data, a weighted food diary could be employed, although this might increase the burden of participation and affect drop-out rates. Another limitation is that supplements were not included in our analyses. However, due to the difficulties in collecting accurate doses from a diverse range of supplement brands, we were not able to quantify the intake of supplements. In addition, it is important to keep in mind the risk of false findings due to multiple testing.

## 5. Conclusions

Maternal intake of cow’s milk during lactation, as confirmed by measurements of dietary biomarkers in maternal blood and breast milk samples, is associated with lower prevalence of physician-diagnosed food allergy by 12 months of age. Furthermore, we show that consumption of fruit and berries during lactation is associated with a higher prevalence of atopic eczema, although this remains to be confirmed with dietary biomarkers. Our results suggest that maternal diet modulate the infant’s immune system, affecting subsequent allergy development.

## Figures and Tables

**Table 1:** Characteristics of children with food allergy, atopic eczema, and asthma, as compared to non-allergic children (i.e., non-allergic, non-sensitized, and non-asthmatic) (*n* = 508).

Variables	Number (%) within Diagnostic Group			
	Non-Allergic (*n* = 389)	Food Allergy (*n* = 39)	Atopic Eczema (*n* = 33)	Asthma (*n* = 33)
Age at delivery (years)				
<25	43 (11)	4 (10)	4 (12)	5 (15)
26–30	176 (45)	17 (44)	14 (42)	10 (30)
31–35	106 (27)	14 (36)	9 (27)	14 (42)
>35	64 (16)	4 (10)	6 (18)	4 (12)
Highest education level				
Elementary school (9 years)	9 (2)	1 (3)	0 (0)	0 (0)
Senior high school (12 years)	99 (26)	12 (31)	12 (36)	15 (47)
University/other education (>12 years)	278 (72)	26 (67)	21 (64)	17 (53)
Missing	3	-	-	1
Maternal nationality				
Swedish	366 (95)	38 (97)	33 (100)	32 (97)
Other	21 (5)	1 (3)	0 (0)	1 (3)
Missing	2	-	-	-
Maternal smoking before pregnancy	22 (6)	1 (3)	0 (0)	2 (6)
Missing	4	-	-	1
Paternal smoking during pregnancy	6 (2)	2 (7)	1 (4)	3 (14) *
Missing	99	11	5	12
Parity				
No previous child	193 (50)	21 (54)	17 (52)	11 (34)
≥1 previous child	194 (50)	18 (46)	16 (48)	21 (66)
Missing	2	-	-	1
≥1 older sibling at home full time	190 (49)	19 (49)	16 (48)	22 (67) *
Early pregnancy BMI				
Underweight (<18.5)	5 (1)	0 (0)	0 (0)	0 (0)
Normal weight (18.5–24.9)	216 (57)	20 (53)	19 (59)	21 (64)
Overweight (25–29.9)	110 (29)	9 (24)	10 (31)	8 (24)
Obese (≥30)	48 (13)	9 (24)	3 (9)	4 (12)
Missing	10	1	1	-
Residential address				
Town (central part)	178 (46)	16 (42)	14 (42)	15 (45)
Town (suburb)	94 (24)	14 (37)	7 (21)	9 (27)
Countryside	112 (29)	8 (21)	12 (36)	9 (27)
Missing	5	1	-	-
Pet ownership (first year of life)				
Dog	125 (32)	8 (21)	7 (21)	9 (27)
Cat	90 (23)	11 (28)	6 (18)	7 (21)
Other	20 (5)	0 (0)	1 (3)	1 (3)
Allergic heredity				
Maternal	143 (37)	23 (59) **	21 (64) **	16 (48)
Paternal	165 (42)	20 (51)	21 (64) *	12 (36)
Sibling	63 (16)	12 (31) **	8 (24)	11 (33)
Any	260 (67)	33 (85) *	32 (97) ***	24 (73)
Season of birth				
Bright season	211 (55)	19 (49)	13 (39)	20 (63)
Dark season	176 (45)	20 (51)	20 (61)	12 (38)
Missing	2	-	-	1
Gender (boy)	170 (44)	24 (62) *	17 (52)	17 (52)
Birthweight in grams				
<2.500	11 (3)	1 (3)	1 (3)	2 (6)
2.500–4.500	359 (93)	37 (95)	31 (94)	25 (78) *
>4.500	17 (4)	1 (3)	1 (3)	5 (16) *
Missing	2	-	-	1
Gestational length				
Pre-term	16 (4)	1 (3)	1 (3)	2 (6)
Term	340 (88)	34 (87)	29 (88)	26 (81)
Post-term	32 (8)	4 (10)	3 (9)	4 (13)
Missing	1	-	-	1
Birth mode				
Vaginal delivery	339 (87)	33 (85)	27 (82)	27 (82)
Cesarean section	50 (13)	6 (15)	6 (18)	6 (18)
Lactation				
Never	18 (5)	2 (6)	2 (6)	0 (0)
<4 months	35 (10)	5 (14)	4 (12)	9 (30) **
4–5 months	56 (15)	8 (22)	6 (18)	8 (27)
≥6 months	253 (70)	21 (58)	21 (64)	13 (43) **
Missing	27	3	-	3

*p*-values for differences between non-allergic group (i.e., non-allergic, non-sensitized, and non-asthmatic children) and the different diagnostic groups are denoted as follows: *, *p* < 0.05; **, *p* < 0.01; ***, *p* < 0.001 (Fisher’s exact test and Pearson’s Chi-square test).

**Table 2:** Dietary intake levels of food items during pregnancy and lactation.

Variables	Food Intake in Gram per Day, Median (25th–75th Percentile)		
	Pregnancy Gestational Week 34 *n* = 488	Lactation 1 Month Postpartum *n* = 430	Lactation 4 Months Postpartum *n* = 397
Bread, total	57 (38–85)	57 (38–83)	53 (30–82)
Cheese	23 (3.9–45)	23 (3.9–45)	22 (3.9–49)
Cow’s milk	100 (14–400)	100 (0–200)	43 (0–200)
Dairy products	310 (190–520)	270 (150–430)	230 (110–390)
Egg	11 (3.6–11)	3.6 (3.6–11)	11 (3.6–25)
Fatty fish	9.1 (6.6–20)	9.1 (6.6–20)	11 (6.6–20)
Fruit and berries	270 (170–410)	200 (120–310)	170 (95–300)
Game meat	0 (0–13)	0 (0–18)	0 (0–13)
Grain, fiber	40 (19–72)	38 (16–70)	38 (16–66)
Grain, low fiber	55 (31–85)	57 (32–92)	55 (26–88)
Lean fish	6.6 (6.6–20)	6.6 (4.1–20)	6.6 (6.6–20)
Meat, total	97 (68–130)	100 (70–140)	100 (68–140)
Nuts and seeds	2.9 (0–8.6)	1.7 (0–8.6)	2.9 (0–8.6)
Offal	3.0 (0–9.6)	1.0 (0–9.1)	1.0 (0–7.6)
Pizza	25 (25–25)	25 (25–25)	25 (0–25)
Potato	31 (21–47)	31 (21–54)	31 (21–47)
Poultry	20 (6.6–20)	20 (6.6–20)	20 (6.6–20)
Processed meat	27 (16–40)	30 (18–43)	30 (16–43)
Red meat	53 (36–73)	54 (39–79)	54 (39–79)
Root vegetables	15 (5.5–30)	13 (4.3–30)	18 (6.4–35)
Seafood, total	26 (17–39)	26 (13–38)	26 (18–39)
Shellfish	4.1 (0–6.6)	0 (0–6.6)	0 (0–6.6)
Snacks	4.3 (2.1–6.4)	4.3 (2.1–8.6)	4.3 (2.1–8.6)
Soft drinks	43 (14–100)	43 (14–100)	14 (0–43)
Sweets	40 (20–69)	43 (20–73)	37 (16–60)
Vegetables	120 (58–210)	110 (49–190)	120 (58–200)
Vegetarian dishes	18 (0–24)	9.1 (0–24)	18 (0–54)
Yoghurt	100 (43–200)	100 (14–200)	100 (7.1–200)

Definition of what is included in each variable is presented in Supplementary Table S1. Energy-adjusted intake levels (gram per MJ) are presented in Supplementary Table S2.

**Table 3:** Dietary intake levels of nutrients during pregnancy and lactation.

Variables	Median (25th–75th Percentile)		
	Pregnancy Gestational Week 34 *n* = 488	Lactation 1 Month Postpartum *n* = 430	Lactation 4 Months Postpartum *n* = 397
Energy, kJ	7200 (5600–8800)	7000 (5500–9200)	6800 (5300–8500)
Energy, kcal	1700 (1300–2100)	1700 (1300–2200)	1600 (1300–2000)
Protein, g	70 (55–86)	69 (55–88)	69 (55–85)
Fat, g	67 (51–84)	68 (53–91)	65 (49–84)
Cholesterol, mg	220 (170–280)	220 (170–290)	230 (180–290)
Monounsaturated fat, g	23 (18–30)	24 (19–32)	24 (18–30)
Polyunsaturated fat, g	8.1 (5.9–11)	8.1 (6.0–11)	8.3 (6.3–11)
Fatty acid 20:5 (EPA), mg	79 (50–150)	75 (52–150)	77 (52–150)
Fatty acid 22:5 (DPA), mg	46 (31–76)	45 (32–74)	46 (32–75)
Fatty acid 22:6 (DHA), mg	170 (110–300)	160 (110–290)	170 (110–300)
Saturated fat, g	30 (22–37)	29 (22–40)	27 (21–37)
Trans fat, g	0.75 (0.52–1.0)	0.76 (0.53–1.1)	0.71 (0.45–1.0)
Carbohydrates, g	200 (150–240)	190 (140–250)	180 (130–230)
Disaccharides, g	61 (45–82)	59 (43–83)	54 (40–74)
Monosaccharides, g	32 (22–43)	28 (20–38)	26 (18–35)
Fiber, g	18 (12–25)	17 (11–25)	17 (12–24)
Sucrose, g	36 (25–52)	35 (23–51)	30 (21–44)
Whole grain, g	34 (16–63)	38 (18–62)	40 (20–61)
Alpha-Carotene, μg	2500 (1500–4100)	2300 (1400–4000)	2700 (1600–4200)
Calcium, mg	1000 (760–1300)	1000 (760-1400)	1000 (750–1300)
Folate, μg	290 (220–380)	280 (200–360)	280 (210–350)
Iodine, μg	99 (69–140)	110 (69–150)	110 (76–150)
Iron, mg	10 (7.1–13)	10 (7.1–14)	9.5 (7.1–13)
Magnesium, mg	290 (220–380)	300 (210–380)	290 (230–370)
Niacin, mg	15 (11–19)	15 (11–19)	15 (12–19)
Phosphorus, mg	1400 (1000–1700)	1300 (1000–1700)	1300 (1100–1600)
Potassium, mg	2900 (2200–3400)	2800 (2100–3600)	2800 (2200–3400)
Retinol, μg	380 (270–530)	370 (260–530)	350 (240–480)
Riboflavin, mg	1.7 (1.2–2.1)	1.6 (1.2–2.2)	1.6 (1.2–2.0)
Selenium, μg	40 (30–51)	38 (29–52)	39 (30–54)
Sodium, mg	2200 (1700–2800)	2200 (1800–2800)	2300 (1800–2800)
Thiamine, mg	1.2 (0.93–1.6)	1.2 (0.87–1.6)	1.1 (0.92–1.5)
Vitamin A, μg	650 (480–850)	640 (450–860)	640 (470–870)
Vitamin B12, μg	4.9 (3.6–6.5)	4.9 (3.6–6.6)	4.9 (3.8–6.2)
Vitamin B6, mg	1.7 (1.3–2.1)	1.6 (1.2–2.0)	1.6 (1.2–2.0)
Vitamin C, mg	97 (63–140)	78 (53–110)	74 (49–110)
Vitamin D, μg	6.3 (4.2–8.8)	6.3 (4.5–8.8)	6.6 (4.8–8.7)
Vitamin E, mg	8.0 (6.1–10)	7.8 (5.9–10)	8.4 (6.3–11)
Vitamin K, μg	30 (22–40)	30 (20–40)	31 (21–40)
Zinc, mg	9.3 (7.1–12)	9.5 (7.4–12)	9.2 (7.5–11)

Dietary intake levels of nutrients are based on nutritional calculations, not including supplements. Energy-adjusted intake levels (per MJ) are presented in Supplementary Table S3.

**Table 4:** Correlations between maternal dairy intake and fatty acid proportions in breast milk and in maternal erythrocytes.

Variables	Breast Milk				Erythrocytes			
	1 Month		4 Months		Pregnancy		4 Months	
	Rho	*p*	Rho	*p*	Rho	*p*	Rho	*p*
**Dairy products**								
Pentadecanoic acid (15:0)	0.229	<0.001	0.328	<0.001	0.194	<0.001	0.272	<0.001
Heptadecanoic acid (17:0)	0.161	0.006	0.287	<0.001	-	NS ^1^	0.144	0.021
**Cow’s milk**								
Pentadecanoic acid (15:0)	0.192	<0.001	0.251	<0.001	0.152	<0.001	0.170	0.006
Heptadecanoic acid (17:0)	0.167	0.004	0.238	<0.001	-	NS	0.132	0.034
**Cheese**								
Pentadecanoic acid (15:0)	0.284	<0.001	0.278	<0.001	0.158	<0.001	0.266	<0.001
Heptadecanoic acid (17:0)	0.219	<0.001	0.154	0.009	-	NS	0.140	0.025

The proportions of fatty acids were analyzed in maternal breast milk obtained 1 (*n* = 297) and 4 months (*n* = 284) postpartum, and in maternal erythrocytes obtained from blood sampled during pregnancy (Week 28, *n* = 476) and lactation (4 months postpartum, *n* = 257). Associations to maternal dietary intake in gram per day were tested with Spearman’s correlation test. Definition of what is included in each dietary variable is presented in Supplementary Table S1. ^1^ Not significant.

## Data Availability

The food frequency questionnaires are not publicly available due to proprietary rights. The raw data used in this study are not publicly available because they relate to information that could compromise research participant privacy or consent. Explicit consent to deposit raw data was not obtained from the participants. Therefore, the data can only be made public if a new consent is filled in by the participants together with a new ethical permit being obtained. The R-code for conducting the partial Spearman correlation and the clustered heatmaps can be obtained from: https://gitlab.com/miastravik/maternal-diet-in-relation-to-offspring-allergy.

## Supplementary Materials

The following are available online at https://www.mdpi.com/2072-6643/12/12/3680/s1, Supplementary Materials, Table S1. Food groups included in the statistical analyses, Table S2. Dietary intake levels (gram per MJ) of food items during pregnancy and lactation, Table S3. Dietary intake levels of nutrients (per MJ) during pregnancy and lactation, Table S4. Significant confounder adjusted correlations between maternal food intake and allergy in offspring, Table S5. Significant crude correlations between maternal food intake and allergy in offspring, Table S6. Significant confounder adjusted correlations between maternal nutrient intake and offspring allergy, Table S7. Significant crude correlations between maternal nutrient intake and allergy in offspring.
