# Supplementary material for: Maternal Intake of Cow’s Milk during Lactation Is Associated with Lower Prevalence of Food Allergy in Offspring
Source: Nutrients. 2020 Nov 28;12(12):3680. doi: 10.3390/nu12123680 (PMC7761074; doi:10.3390/nu12123680)
Supplement: Supplementary file 1 [file nutrients-12-03680-s001.pdf]

Supplementary Materials

Analysis of fatty acids in erythrocytes

The total fatty acid composition was assessed in maternal blood cells using the gas chromatography with flame ionization detection (GC–FID) method after the extraction of fatty acids that were converted to methyl esters according to a modified version of the method described previously [1]. EDTA-treated blood samples were collected from the mothers at Gestational Week 28 and at 4 months postpartum. The samples were centrifuged, the plasma was removed, and the remaining blood, hereinafter referred to as "erythrocytes" due to the negligible number of white blood cells, was frozen at −80 °C. For methylation, 50 μL of erythrocytes were mixed with 10 μg of internal standard (methyl tricosanoate, C23:0 methyl ester), followed by the addition of 1.8 mL of acetyl chloride-MeOH solution 10% (*v/v*) fortified with butylated hydroxytoluene (2.78 μg/mL) and incubated at 70 °C for 60 min in a water bath. A single extraction of the fatty acid methyl esters was carried out by adding 1.5 mL of n-hexane, which was then evaporated at 125 mbar and 30 °C for 30 min. The samples were dissolved in 200 μL hexane and injected into the GC-FID for analysis, with each batch consisting of 50 study samples, two water blanks, and six quality controls prepared with Millipore-purified water (Merck KGaA, Darmstadt, Germany). A pool of erythrocytes from five donors was aliquoted and stored at −80°C. This quality control sample was analyzed after every tenth sample to check the performance of the GC-FID system. To prevent oxidation during storage, 0.75 mL of RBC 30 μL of BHT solution (0.5 mg BHT/mL MeOH) were added prior to aliquoting. The standard GLC-462 mixed fatty acid methyl esters (Nu-Chek Prep, Elysian, MN, USA) was dissolved in toluene to create an external standard calibration (50, 35, 20, 10, 5 and 2.5 μg/mL). C23:0 dissolved in toluene was used as an internal standard in all the samples, as well as in the external standard used for derivation of the calibration curve.

The fatty acids were separated on the GC-FID system that consisted of: the Thermo Fisher Scientific Al/AS 1310 TRACE 1300 series GC-mainframe, FID detector, Thermo Fisher Scientific Al 1310 autosampler, Thermo Fisher Scientific NM Plus H2 generator, together with a MicroClip XT hydrogen gas alert and a ZA 1500 zero air generator. The GC-FID column was the Zebron ZB-FAME (20 m × 0.18 μm ID × 0.15 μm phase) (P/N 7FD-G033-05; Phenomenex Inc., Torrance, CA, USA). The liner was the Phenomenex Zebron Plus, 4-mm ID, with Single Taper Wool on Bottom (P/N AG2- OA11-05). Also used were the Agilent Ultra Inert Inlet Liner, Low PSI drop, wool (P/N 5190-2295) and septa from Thermo Fisher Scientific, BTO-coated, 11 mm (P/N: 31303233).

The following oven program was applied: initial 80 °C with a 1.5-min hold; ramping at 40 °C/min to 160 °C; 5 °C/min to 185 °C with 0-min hold, and then 30 °C/min to 260 °C with 0-min hold. The instrumental conditions were: H_2 as carrier gas, constant flow, carrier flow rate of 1.25 mL/min, inlet temperature of 260 °C, split flow at 12.5 mL/min, and split ratio of 15. The detector temperature was 260 °C. The gas flows were: air, 450 mL/min; hydrogen, 35 mL/min; and makeup gas, 10 mL/min. The injection volume was 1 μL per sample, with an oven run time of 13 min and an overall sample-to- sample run time of 18 min. Each run consisted of 68 samples: 1 hexane sample, external calibration standards at 6 levels, 1 blank, 1 QC, 10 study samples (repeated 4 times), 1 QC, 1 blank, 1 external standard, 1 external calibration standard level 2, and 1 hexane.

Data were acquired using the Thermo Fisher Scientific Xcalibur ver. 4.3 software. Twenty-two fatty acids were quantified in each sample: 14:0, 15:0, 16:0, 16:1 n-7, 17:0, 18:0, 18:1 n-7, 18:1 n-9, 18:2 n-6, 18:3 n-3, 20:0, 20:1 n-11, 20:2 n-9, 20:3 n-6, 20:4 n-6, 20:5 n-3, 22:0, 22:4 n-6, 22:5 n-3, 22:6 n-3, 24:0, 24:1 n-9. The concentration of each fatty acid was calculated using a standard curve of the external standard with the internal standard C23:0 added. The proportion of specific fatty acids is expressed as the area of the particular fatty acid, relative to the concentration of all 22 fatty acids.

Analysis of fatty acids in breast milk

The concentrations of fatty acids in the total lipid fraction of breast milk samples were analyzed by gas chromatography after conversion to methyl esters [2]. Breast milk samples (100 µL) were thawed slowly in cold water, vortexed, and mixed with 50 µL of internal standard (fatty acid 19:0, 1 mg/mL), 1 mL toluene, and 1 mL acetyl chloride (10%) dissolved in methanol. After incubation at 70 °C for 2 h, the methyl esters were extracted with 1 mL petroleum ether and 1 mL MilliQ-water. After evaporation under a nitrogen flow at 40 °C, the samples were dissolved in 1 mL isooctane and separated in a gas chromatography-mass spectrometry system (5975C; Agilent Technologies Inc., Santa Clara, CA, USA). A pool of breast milk from four donors was aliquoted and stored at −80 °C. This quality control sample was analyzed after every tenth sample to control the performance of the GC-MS system. The standard set of GLC-463 mixed fatty acid methyl esters (Nu-Chek Prep) was dissolved in toluene and used as an external standard for peak evaluation.

The fatty acids were separated on the GC-MS system that consisted of the Agilent Technologies 7890A GC-system, Agilent Technologies 5975C inert XL EI/CI MSD with Triple-Axis Detector, and the Agilent Technologies 7693 Autosampler. The GC column used was the VF-WAXms (30 m × 0.25 mm × 0.25 µm) (P/N 7FD-G033-05; Agilent Technologies). The liner was the Ultra Inert Inlet Liner, Low PSI drop, wool (P/N 5190-3165; Agilent Technologies). The Thermo Fisher Scientific Bleed/Temp Optimized Non-Stick 11-mm Septa was employed.

The GC oven program consisted of: initial 100 °C, then ramping at 4 °C/min to 205 °C and then at 1 °C/min to 230 °C with a 5-min hold. The instrumental conditions were: helium as the carrier gas; inlet heater at 275 °C; pressure, 10.523 psi; total flow, 14 mL/min, septum purge flow, 3 mL/min; split flow, 10 mL/min; and split ratio, 10. The injection volume was 1 µL per sample, with an oven run time of 56 min.

Data were acquired using the Agilent MassHunter™ software. Thirty-three fatty acids were quantified in each sample: 10:0, 12:0, 14:0, 15:0, 16:0, 17:0, 18:0, 20:0, 22:0, 24:0, 14:1 n-5, 15:1, 16:1 n-7, 17:1 n-7, 18:1 n-7, 18:1 n-9, 19:1 n-9, 20:1 n-9, 22:1 n-9, 20:1 n-15, 18:3 n-3, 20:3 n-3, 20:5 n-3, 22:3 n-3, 2:5 n-3, 22:6 n-3, 18:2 n-6, 18:3 n-6, 20:2 n-6, 20:3 n-6, 20:4 n-6, 22:2 n-6, and 22:4 n-6. The concentration of each fatty acid was calculated using the concentration of the internal standard 19:0. The proportions of specific fatty acids were expressed as the concentration of the particular fatty acid relative to the concentration of all 33 fatty acids.

References

1. Masood, A.; Stark, K.D.; Salem, N. A simplified and efficient method for the analysis of fatty acid methyl esters suitable for large clinical studies. *J. Lipid Res.* **2005**, *46*, 2299–2305, doi:10.1194/jlr.d500022-jlr200.
2. Lepage, G.; Roy, C.C. Improved recovery of fatty acid through direct transesterification without prior extraction or purification. *J. Lipid Res.* 1984, *25*.

Table S1. Food groups included in the statistical analyses. All variables are reported in gram per day, estimated based on the reported intake frequency and amount.

	**Asked for in the FFQ**
**Bread, total**	Crispbread; White bread e.g., sliced bread, bread loaf or flatbread; Soft grain bread (e.g., rye bun, whole grain bread or dark rye bread)
**Cheese**	Hard cheese; Cottage cheese, dessert cheese (e.g., Philadelphia cream cheese, brie)
**Cow's milk**	Cow's milk in glass or on plate
**Dairy products**	Cow's milk (see above); Yoghurt (see below); Cheese (see above); Cream sauce, crème fraiche or sour cream sauce; Chocolate cow's milk
**Egg**	Egg or omelet
**Fatty fish**	Salmon, sushi, Baltic herring, Atlantic herring or mackerel; Tuna
**Fruit and berries**	Banana; Apple or pear; Orange, Small citrus or grapefruit; Other fruit; Fresh or frozen berries; Dried fruits and berries; Juice; Fruit drink, fruit soup, kissel, smoothie; Marmalade, jam, apple sauce, honey*
**Game meat**	Reindeer; Moose; Deer; Wild boar; Hare, grouse, Anseriformes, pheasants or other smaller game meat species
**Grain, fiber**	Whole grain pasta; Brown rice; Soft grain bread (e.g., rye bun, whole grain bread or dark rye bread); Crispbread
**Grain, low fiber**	Refined pasta or noodles; Rice; White bread (e.g., sliced bread, bread loaf or flatbread)
**Lean fish**	White fish (cod, saithe, fish sticks, fish balls)
**Meat, total**	Red meat (see below); Game meat (see above); Processed meat (see below); Offal (see below)
**Nuts and seeds**	Nuts, almonds or seeds; Flaxseed
**Offal**	Liver paste; Blood pudding or blood sausage; Liver or kidney
**Pizza**	Pizza, pie or pasty
**Potato**	French fries, fried potato, *pyttipanna* (Swedish hash), potato gratin; Boiled potato, mashed potato or baked potato
**Poultry**	Chicken or other poultry (e.g., fried, boiled, deep-fried or in a stew)
**Processed meat**	Sausage; Bacon, roast pork or pork belly; Meat-based spreads (e.g., ham or salami)
**Red meat**	Ground-meat dishes (e.g., meat sauce, lasagna or meatballs); beef (e.g., steak, filler or stew); Hamburger, kebab or tacos; Pork (e.g., pork chop, fillet or stew); Lamb or game
**Root vegetables**	Carrots; beetroot, parsnip, Swedish turnip, celeriac
**Seafood, total**	Fatty fish (see above); Lean fish (see above); Shellfish (see below)
**Shellfish**	Shellfish (e.g., shrimps or mussels)
**Snacks**	Popcorn; Crisps or cheese puffs; Biscuits or cookies
**Soft drinks**	Soft drink, cider or squash (drink)
**Sweets**	Candy (not chocolate); Chocolate; Ice cream or parfait; Bun, muffin or sponge cake; Pastry, cake or sweet pie
**Vegetables**	Salad meals; Vegetable mix; Tomato or bell pepper; Lettuce, cucumber, squash, zucchini; Spinach or arugula; Corn; Broccoli or cabbage; Green peas or beans; Beans, lentils or hummus; Onion
**Vegetarian dishes**	Vegetarian vegetable dishes (e.g., gratin, lasagna or wok); Vegetarian protein-rich dishes (e.g., bean burger, soy sausage or Quorn)
**Yoghurt**	Fil (i.e., fermented cow's milk product), yoghurt or yoghurt drink

*Could not be separated from marmalade, jam and apple sauce owing to the question design.

Table S2. Dietary intake levels (gram per MJ) of food items during pregnancy and lactation.

Food intake in gram per day, median (25th– 75th percentile)			
	**Pregnancy Gestational Week 34 n=488**	**Lactation 1 month postpartum n=430**	**Lactation 4 months postpartum n=397**
Bread, total	8.4 (5.4–12)	8.4 (5.9–11)	8.2 (4.6–12)
Cheese	3.0 (0.56–5.8)	3.1 (1.0–6.0)	2.8 (0.45–5.7)
Cow's milk	15 (2.1–39)	13 (0–30)	6.6 (0–26)
Dairy products	44 (28–67)	37 (24–58)	33 (18–52)
Egg	1.0 (0.45–2.1)	0.82 (0.35–1.8)	1.2 (0.43–3.3)
Fatty fish	1.6 (0.81–3.0)	1.6 (0.88–2.9)	1.8 (0.92–3.2)
Fruit and berries	39 (25–59)	28 (17–45)	25 (14–44)
Game meat	0 (0–1.8)	0 (0–1.9)	0 (0–2.1)
Grain, fiber	6.0 (3.0–10)	5.7 (2.6–9.4)	5.4 (2.6–9.5)
Grain, low fiber	8.1 (4.3–12)	8.6 (4.4–13)	8.0 (4.1–13)
Lean fish	1.3 (0.66–2.3)	1.1 (0.54–2.2)	1.2 (0.72–2.2)
Meat, total	14 (9.7–19)	15 (10–21)	15 (9.9–21)
Nuts and seeds	0.31 (0–1.2)	0.18 (0–1.0)	0.36 (0–1.4)
Offal	0.40 (0–1.3)	0.23 (0–1.2)	0.14 (0–1.2)
Pizza	3.2 (2.1–4.6)	3.4 (2.1–4.9)	3.2 (0–4.7)
Potato	4.2 (2.6–6.8)	4.4 (2.8–7.0)	4.6 (3.0–7.1)
Poultry	2.2 (1.1–3.4)	2.4 (1.1–3.6)	2.4 (1.2–3.7)
Processed meat	3.9 (2.3–5.7)	4.3 (2.5–6.2)	4.3 (2.4–6.6)
Red meat	7.7 (5.2–11)	7.9 (5.1–12)	8.4 (5.4–12)
Root vegetables	2.2 (0.83–4.2)	2.0 (0.66–4.2)	2.6 (0.91–4.9)
Seafood, total	4.0 (2.2–5.7)	3.5 (2.2–5.4)	3.8 (2.3–5.9)
Shellfish	0.54 (0–1.1)	0 (0–0.97)	0 (0–1.1)
Snacks	0.57 (0.27–0.98)	0.66 (0.35–1.1)	0.69 (0.35–1.2)
Soft drinks	5.4 (1.7–12)	4.8 (1.1–12)	3.7 (0–9.2)
Sweets	5.8 (2.9–9.3)	6.3 (3.0–9.6)	5.4 (2.7–8.2)
Vegetables	18 (8.5–29)	16 (6.9–27)	18 (8.4–30)
Vegetarian dishes	1.9 (0–4.1)	1.3 (0–4.1)	2.2 (0–6.3)
Yoghurt	16 (5.6–27)	14 (3.3–22)	11 (0.58–23)

Definition of what is included in each variable is presented in Supplementary Table S1.

Table S3. Dietary intake levels of nutrients (per MJ) during pregnancy and lactation.

	Median (25th– 75th percentile)		
	**Pregnancy Gestational Week 34 n=488**	**Lactation 1 month postpartum n=430**	**Lactation 4 months postpartum n=397**
Protein, g	9.7 (8.8-11)	9.8 (8.7-11)	10 (8.9-11)
Fat, g	9.5 (8.8-10)	9.8 (8.9-11)	9.8 (9.1-11)
Cholesterol, mg	31 (26-36)	31 (26-36)	32 (27-39)
Monounsaturated fat, g	3.4 (3.0-3.7)	3.5 (3.1-3.8)	3.5 (3.2-3.8)
Polyunsaturated fat, g	1.1 (0.92-1.4)	1.1 (0.95-1.4)	1.2 (1.0-1.5)
Fatty acid 20:5 (EPA), mg	12 (6.9-20)	11 (7.2-20)	13 (7.3-22)
Fatty acid 22:5 (DPA), mg	6.8 (4.4-10)	6.7 (4.5-9.8)	7.1 (4.7-11)
Fatty acid 22:6 (DHA), mg	26 (15-39)	24 (16-40)	27 (16-45)
Saturated fat, g	4.1 (3.7-4.6)	4.3 (3.8-4.8)	4.2 (3.6-4.7)
Trans fat, g	0.11 (0.085-0.13)	0.11 (0.089-0.13)	0.10 (0.079-0.13)
Carbohydrates, g	27 (25-29)	26 (24-29)	26 (24-28)
Disaccharides, g	8.9 (7.2-10)	8.6 (6.8-11)	8.1 (6.7-10)
Monosaccharides, g	4.5 (3.6-5.6)	3.8 (3.0-4.9)	3.7 (2.8-4.9)
Fiber, g	2.5 (1.9-3.2)	2.4 (1.8-3.1)	2.5 (1.9-3.2)
Sucrose, g	5.3 (3.9-6.8)	4.9 (3.7-6.9)	4.6 (3.3-5.9)
Whole grain, g	5.2 (2.7-7.8)	5.3 (2.7-8.2)	5.7 (3.0-8.5)
Alpha-Carotene, μg	360 (220-560)	330 (200-530)	400 (240-590)
Calcium, mg	140 (120-170)	140 (120-170)	140 (120-180)
Folate, μg	42 (35-49)	39 (33-46)	40 (34-49)
Iodine, μg	14 (10-18)	14 (10-19)	15 (12-21)
Iron, mg	1.4 (1.2-1.7)	1.4 (1.1-1.7)	1.4 (1.2-1.7)
Magnesium, mg	41 (37-46)	41 (36-47)	43 (37-49)
Niacin, mg	2.1 (1.8-2.4)	2.1 (1.8-2.4)	2.2 (1.9-2.6)
Phosphorus, mg	190 (170-210)	190 (160-210)	190 (170-220)
Potassium, mg	390 (350-450)	380 (340-440)	400 (350-450)
Retinol, μg	53 (42-67)	51 (40-65)	51 (40-64)
Riboflavin, mg	0.23 (0.20-0.27)	0.22 (0.19-0.26)	0.23 (0.20-0.27)
Selenium, μg	5.5 (4.5-7.1)	5.3 (4.4-6.8)	5.9 (4.6-7.4)
Sodium, mg	310 (280-350)	320 (280-360)	330 (290-380)
Thiamine, mg	0.18 (0.15-0.20)	0.17 (0.14-0.19)	0.17 (0.15-0.20)
Vitamin A, μg	90 (74-110)	86 (71-110)	93 (76-110)
Vitamin B12, μg	0.70 (0.56-0.86)	0.69 (0.56-0.85)	0.73 (0.59-0.89)
Vitamin B6, mg	0.24 (0.20-0.27)	0.23 (0.19-0.26)	0.23 (0.20-0.27)
Vitamin C, mg	14 (9.5-19)	11 (7.5-16)	11 (7.2-16)
Vitamin D, μg	0.86 (0.66-1.2)	0.90 (0.68-1.1)	0.94 (0.73-1.2)
Vitamin E, mg	1.1 (0.95-1.4)	1.1 (0.92-1.3)	1.2 (0.98-1.5)
Vitamin K, μg	4.2 (3.2-5.4)	4.0 (3.0-5.3)	4.3 (3.4-6.0)
Zinc, mg	1.3 (1.2-1.4)	1.3 (1.2-1.5)	1.4 (1.2-1.5)

Dietary intake levels of nutrients are based on nutritional calculations, not including supplements.

Table S4. Significant confounder-adjusted correlations between maternal food intake and allergy in. offspring.

	Food allergy		Atopic eczema		Asthma	
**Pregnancy[1]**	Rho_{adj}	*P*adj	Rho_{adj}	*P*adj	Rho_{adj}	*P*adj
Cheese	-0.105	0.034	-	-	-	-
Game meat	-	-	-	-	-0.134	0.008
Poultry	0.113	0.023	-	-	-	-
**1 month postpartum[2]**						
Cow's milk	-0.118	0.025	-	-	-	-
Red meat	-	-	-	-	-0.122	0.022
Vegetarian dishes	-	-	-	-	-0.120	0.024
**4 months postpartum[3]**						
Cow's milk	-0.186	<0.001	-	-	-	-
Dairy products	-0.162	0.003	-	-	-	-
Fruit & berries	-	-	0.182	<0.001	-	-
Game meat	-	-	-	-	-0.129	0.019
Nuts & seeds	-	-	0.139	0.012	-	-
Pizza	-0.151	0.006	-	-	-	-
Processed meat	-	-	-	-	-0.135	0.015

Associations between offspring allergy and maternal intake of food items (gram per day) were analyzed with partial Spearman's correlations and adjusted for any allergy within family, siblings, season of birth and total energy intake.[1] n=38 with food allergy, n=32 with atopic eczema, and n=31 with asthma. [2] n=34 with food allergy, n=31 with atopic eczema, and n=26 with asthma. [3] n=30 with food allergy, n=27 with atopic eczema, and n=24 with asthma.

Table S5. Significant crude correlations between maternal food intake and allergy in offspring.

	**Food allergy**		**Atopic eczema**		**Asthma**	
**Pregnancy**[1]	Rho_{adj}	*P*adj	Rho_{adj}	*P*adj	Rho_{adj}	*P*adj
Cow's milk	-0.102	0.039	-	-	-	-
Cheese	-0.119	0.016	-	-	-	-
Game meat	-	-	-	-	-0.114	0.022
Poultry	0.102	0.040	-	-	-	-
Sweets	-0.104	0.035	-	-	-	-
**1 month postpartum**[2]						
Cow's milk	-0.139	0.008	-	-	-	-
Dairy products	-0.127	0.015	-	-	-0.115	0.029
Cheese	-	-	-	-	-0.125	0.018
Red meat	-	-	-	-	-0.123	0.020
Vegetarian dishes	-	-	-	-	-0.136	0.010
**4 months postpartum**[3]						
Cow's milk	-0.186	<0.001	-	-	-	-
Dairy products	-0.191	<0.001	-	-	-	-
Yoghurt	-0.136	0.012	-	-	-	-
Lean fish	-0.119	0.029	-0.120	0.028	-	-
Fruit & berries	-	-	0.152	0.005	-	-
Cheese	-0.146	0.007	-0.115	0.035	-	-
Game meat	-	-	-	-	-0.110	0.045
Pizza	-0.190	<0.001	-	-	-	-
Processed meat	-	-	-	-	-0.141	0.010
Potato	-0.125	0.022	-	-	-	-
Total meat	-	-	-	-	-0.112	0.041

Associations between offspring allergy and maternal intake of food items (gram per day) were analyzed with Spearman's correlations. [1] n=38 with food allergy, n=32 with atopic eczema, and n=32 with asthma. [2] n=34 with food allergy, n=31 with atopic eczema, and n=27 with asthma. [3] n=30 with food allergy, n=27 with atopic eczema, and n=25 with asthma.

Table S6. Significant confounder-adjusted correlations between maternal nutrient intake and offspring.allergy.

	Food allergy		Atopic eczema		Asthma	
**1 month postpartum**[1]	Rho_{adj}	*P*adj	Rho_{adj}	*P*adj	Rho_{adj}	*P*adj
Calcium	-	-	-	-	-0.126	0.017
Iodine	-	-	-	-	-0.115	0.030
Phosphorus	-	-	-	-	-0.125	0.018
Vitamin B12	-	-	-	-	-0.105	0.048
Zinc	-	-	-	-	-0.128	0.016
**4 months postpartum**[2]						
Saturated fat	-0.136	0.013	-0.128	0.020	-0.126	0.023
Trans fats	-0.109	0.046	-	-	-	-
Whole grain	0.144	0.008	-	-	-	-
Vitamin B6	0.108	0.048	0.140	0.011	-	-
Vitamin E	0.182	<0.001	0.127	0.020	-	-
Folate	-	-	0.115	0.037	-	-
Monosaccharides	-	-	0.169	0.002	0.112	0.043
Vitamin C	-	-	0.168	0.002	-	-
Total fat	-	-	-	-	-0.156	0.005
Monounsaturated fat	-	-	-	-	-0.141	0.010
Phosphorus	-	-	-	-	-0.121	0.028
Protein	-	-	-	-	-0.112	0.042
Sucrose	-	-	-	-	0.127	0.021

Associations between offspring allergy and maternal intake of nutrients were analyzed with partial Spearman's correlations and adjusted for any allergy within family, siblings, season of birth and total energy intake. [1] n=34 with food allergy, n=31 with atopic eczema, and n=26 with asthma. [2] n=30 with food allergy, n=27 with atopic eczema, and n=24 with asthma.

Table S7. Significant crude correlations between maternal nutrient intake and allergy in offspring.

	**Food allergy**		**Atopic eczema**		**Asthma**	
**1 month postpartum**[1]	**Rho$_{adj}$**	***P*adj**	**Rho$_{adj}$**	***P*adj**	**Rho$_{adj}$**	***P*adj**
Calcium	-0.138	0.008	-	-	-0.168	0.001
Total carbohydrates	-	-	-	-	-0.123	0.020
Disaccharides	-0.130	0.013	-	-	-0.117	0.026
Total fat	-0.120	0.021	-	-	-0.135	0.010
Folate	-0.115	0.028	-	-	-	-
Magnesium	-0.103	0.050	-	-	-0.154	0.003
Monounsaturated fat	-0.105	0.046	-	-	-0.124	0.019
Phosphorus	-0.121	0.021	-	-	-0.167	0.002
Polyunsaturated fat	-0.120	0.022	-	-	-0.121	0.022
Potassium	-0.127	0.015	-	-	-0.157	0.003
Riboflavin	-0.124	0.018	-	-	-0.158	0.003
Saturated fat	-0.115	0.028	-	-	-0.149	0.005
Total protein	-	-	-	-	-0.154	0.003
Vitamin A	-0.114	0.029	-	-	-	-
Vitamin B12	-0.127	0.015	-	-	-0.156	0.003
Vitamin K	-0.114	0.029	-	-	-	-
Iodine	-	-	-	-	-0.161	0.002
Iron	-	-	-	-	-0.104	0.048
Niacin	-	-	-	-	-0.140	0.008
Selenium	-	-	-	-	-0.121	0.022
Sodium	-	-	-	-	-0.131	0.013
Thiamine	-	-	-	-	-0.142	0.007
Trans fat	-	-	-	-	-0.145	0.006
Zinc	-	-	-	-	-0.173	<0.001
**4 months postpartum** [2]						
Calcium	-0.135	0.013	-	-	-	-
Total carbohydrates	-0.110	0.043	-	-	-	-
Disaccharides	-0.139	0.011	-	-	-	-
Total fat	-0.153	0.005	-0.117	0.032	-	-
Magnesium	-0.111	0.041	-	-	-	-
Monounsaturated fat	-0.130	0.017	-	-	-	-
Phosphorus	-0.157	0.004	-	-	-	-
Potassium	-0.151	0.006	-	-	-	-
Protein	-0.148	0.006	-	-	-0.110	0.046
Saturated fat	-0.181	<0.001	-0.134	0.014	-0.109	0.046
Vitamin C	-	-	0.126	0.022	-	-
Vitamin B12	-	-	-	-	-0.112	0.041
Trans fat	-0.168	0.002	-0.122	0.025	-	-
Thiamine	-0.107	0.048	-	-	-	-
Zinc	-0.147	0.007	-	-	-	-

Associations between offspring allergy and maternal intake of nutrients were analyzed with Spearman's correlations. [1] n=34 with food allergy, n=31 with atopic eczema, and n=27 with asthma. [2] n=30 with food allergy, n=27 with atopic eczema, and n=25 with asthma.
